# Supplementary material for: Isolation of Bacterial and Fungal Microbiota Associated with Hermetia illucens Larvae Reveals Novel Insights into Entomopathogenicity
Source: Microorganisms. 2022 Jan 29;10(2):319. doi: 10.3390/microorganisms10020319 (PMC8878651; doi:10.3390/microorganisms10020319)
Supplement: Supplementary file 1 [file microorganisms-10-00319-s001.zip › microorganisms-1537316-supplementary.pdf]

Microorganisms

Isolation of bacterial and fungal microbiota associated with *Hermetia illucens* larvae reveals novel insights into entomopathogenicity

Patrick Klüber[1], Stephanie Müller[1], Jonas Schmidt[1], Holger Zorn[1,2] and Martin Rühl[1,2*]

1 Branch for Bioresources, Fraunhofer Institute for Molecular Biology and Applied Ecology (IME), 35392 Giessen, Germany
2 Institute of Food Chemistry and Food Biotechnology, Justus Liebig University Giessen, Heinrich-Buff-Ring 17, 35392 Giessen, Germany
* Correspondence: martin.ruehl@uni-giessen.de; Tel.: +49 641 9934913

Supplementary Table S1 Taxonomic classification of bacterial isolates from the gut of *H. illucens* larvae grown on PKM. For isolates whose sequences matched two database entries, both suggestions were given. Prokaryotic isolates that could only be assigned to the bacteria were listed as *Bacteria* sp.

Phylum	Class	Order	Family	Species	Isolate	Accession number reference sequence	Sequence identity [%]	Accession number isolate	Medium
Actinobacteria	*Actinobacteria*	*Micrococcales*	*Cellulomonadaceae*	*Cellulomonas flavigena*	1	KX527648	100		TSA, LB
					2	KX527648	100		LB
			Dermacoccaceae	*Dermacoccus nishinomiyaensis*		MF952724	100		TSA
			Microbacteriaceae	*Leucobacter denitrificans*		NR_108568	99.90	MZ621119	TSA
				Microbacterium thalassium	1	KF973240	99.33	MZ621120	LB
					2	MG833351	98.99	MZ621121	TSA
			Micrococcaceae	*Kocuria rhizophila*		MN013884	100		TSA
				Micrococcus sp.	1	LC484762	100		LB
					2	MH741267	100		LB
					3	MH741267	100		TSA
			Promicromonosporaceae	*Cellulosimicrobium* sp.		LC496847	99.91	MZ621122	TSA
Bacteroidetes	*Flavobacteriia*	*Flavobacteriales*	*Flavobacteriaceae*	*Empedobacter falsenii*		FM162560	99.61	MZ621123	TSA

				Empedobacter falsenii / Empedobacter brevis		KF254733 / MK087737	100		LB
				Flavobacterium ceti	1	NR_042540	99.62	MZ621124	LB
					2	NR_042540	99.35	MZ621125	TSA
	Sphingobacteriia	*Sphingobacteriales*	*Sphingobacteriaceae*	*Sphingobacterium* sp.		KT260493	98.31	MZ621126	TSA
				Sphingobacterium tabacisoli	1	NR_159136	100		LB
					2	NR_159136	100		TSA
				Sphingobacterium thalpophilum	1	KP326566	100		M_2
					2	LR590484	100		TSA
Firmicutes	*Bacilli*	*Bacillales*	*Bacillaceae*	*Bacillus* sp.		LC484649	100		TSA
				Bacillus infantis / Bacillus drentensis		MK241868 / MF351825	100		TSA
				Bacillus licheniformis / Bacillus paralicheniformis		CP042252 / MG076981	100		TSA
			Paenibacillaceae	*Cohnella* sp.		JN617229	99.34	MZ621127	TSA
				Paenibacillus cineris / Paenibacillus favisporus	1	MK123318 / KT382227	99.67	MZ621128	TSA
					2	LN890143 / JN867753	100		LB
					3	LN890143 / KY622167	100		LB
					4	LN890143 / JN867753	100		LB
					5	LN890143 / JN867753	100		TSA
				Paenibacillus konkukensis / Paenibacillus vulneris		NR_157640 / KP980604	100		TSA
				Paenibacillus vini		MK014238	99.90	MZ621129	LB, TSA
		Lactobacillales	*Enterococcaceae*	*Enterococcus* sp.	1	LC484771	100		TSA
					2	LC473138	100		LB
					3	LC473138	100		TSA
					4	JQ680291	100		TSA
				Enterococcus gallinarum / Enterococcus casseliflavus	1	MK929299 / MK796027	100		LB
					2	MK005268 / MN213357	99.90	MZ621130	LB
					3	MK005268 / MN213357	99.90	MZ621131	TSA
					4	MK005268 / MN213357	100		TSA
					5	MK005268 / MN213357	100		TSA

			Lactobacillaceae	*Lactobacillus plantarum* / *Lactobacillus paraplantarum*		MN326667 / MN255763	100		TSA
Proteobacteria	*Alphaproteobacteria*	*Rhizobiales*	*Brucellaceae*	*Ochrobactrum* sp.		LC484683	100		LB
				Brucella intermedia	1	NR_113812	99.79	MZ621132	TSA
				Bordetella sp.	2	HQ652589	100		TSA, LB
	Betaproteobacteria	*Burkholderiales*	*Alcaligenaceae*	*Achromobacter* sp.		MK602372	100		TSA
				Achromobacter xylosoxidans / *Achromobacter insolitus*		MK537386 / KJ806214	100		TSA
				Alcaligenes faecalis	1	MN176578	99.83	MZ621133	TSA
					2	MN176578	100		LB
					3	MN176578	100		TSA
				Bordetella sp.	1	HQ652589	100		TSA
					2	HQ652589	99.85	MZ621134	TSA
					3	KR188957	99.90	MZ621135	TSA
				Bordetella muralis / *Bordetella petrii*	1	NR_145920 / KP259605	99.91	MZ621136	LB
					2	NR_145920 / KP259605	99.63	MZ621137	TSA, LB
	Gammaproteobacteria	*Enterobacterales*	*Enterobacteriaceae*	*Citrobacter amalonaticus*	1	MH220304	99.61	MZ621138	TSA
					2	MH220304	99.51	MZ621139	TSA, LB
				Klebsiella sp.		MN258617	100		TSA
				Klebsiella oxytoca		LR607350	100		LB
				Klebsiella pneumoniae		CP040363	100		LB
				Klebsiella pneumoniae / *Klebsiella aerogenes*	1	MK764697 / LR134254	100		LB
					2	CP030072 / LR134254	100		TSA
				Klebsiella pneumoniae / *Klebsiella variicola*	1	MH111559 / KJ123840	100		LB
					2	MH111559 / KJ123840	100		LB
		Pseudomonales	*Pseudomonadaceae*	*Pseudomonas* sp.	1	MN173964	100		TSA
					2	MN136109	100		TSA
				Pseudomonas aeruginosa		MN314664	100		TSA
-	-	-	-	*Bacteria* sp.	1	-	-	MZ669995	TSA
					2	-	-	MZ669996	TSA
					3	-	-	MZ669997	LB

Supplementary Table S2 Taxonomy of fungal isolates from the gut of *H. illucens* larvae grown on PKM.
For isolates whose sequences matched two database entries, both suggestions were given.

Phylum	Class	Order	Family	Species	Isolate	Accession number reference sequence	Sequence identity [%]	Accession number isolate	Medium
Ascomycota	*Sordariomycetes*	*Hypocreales*	*Nectriaceae*	*Fusarium solani species complex*		MH370156	100		YPD
				Fusarium sp.	1	MF327378	100		YPD
					2	MH050788	100		M_2
	Saccharomycetes	*Saccharomycetales*	*Debaryomycetaceae*	*Candida tropicalis*	1	KJ451642	99.79	MZ648454	LB
					2	KJ451642	99.75	MZ648455	M_2, YPD
					3	KJ451642	99.78	MZ648456	LB, TSA
			Dipodascaceae	*Sporopachydermia lactativora*		KY105534	99.81	MZ648457	YPD
			Pichiaceae	*Pichia kudriavzevii*	1	MK998698	100		YPD, M_2
					2	MK998698	100		M_2
			Pichiaceae / Debaryomycetaceae	*Pichia kudriavzevii / Suhomyces xylopsoci*	1	MN310535 / MF685426	100		YPD
					2	MN310535 / MF685426	100		M_2
			Saccharomycetaceae	*Diutina rugosa*		KM368818	100		YPD
				Diutina rugosa / Kluyveromyces marxianus	1	KM368818 / MH748688	100		LB
					2	MK394158 / MH748688	100		M_2, YPD
					3	MK394158 / MH748688	100		M_2
					4	MK394158 / MH748688	100		TSA, LB
					5	MK394158 / MH748688	100		TSA
					6	MK394158 / MH748688	100		TSA, LB, M_2
Basidiomycota	*Tremellomycetes*	*Trichosporonales*	*Trichosporonaceae*	*Trichosporon asahii*	1	KT900123	100		YPD
					2	KT900123	100		M_2
					3	MG241533	100		M_2, YPD, TSA
					4	MN318469	100		M_2
					5	MN318469	100		TSA
Mucoromycota	*Mucoromycetes*	*Mucorales*	*Lichtheimiaceae*	*Lichtheimia ramosa*		MF033505	100		M_2
